# The Influence of the *Pinyin* and *Zhuyin* Writing Systems on the Acquisition of Mandarin Word Forms by Native English Speakers

**DOI:** 10.3389/fpsyg.2016.00785

**Published:** 2016-06-03

**Authors:** Rachel Hayes-Harb, Hui-Wen Cheng

**Affiliations:** Department of Linguistics, University of UtahSalt Lake City, UT, USA

**Keywords:** second language acquisition (SLA), mandarin, *Pinyin*, *Zhuyin*, orthographic input, second language phonology, second language word learning

## Abstract

The role of written input in second language (L2) phonological and lexical acquisition has received increased attention in recent years. Here we investigated the influence of two factors that may moderate the influence of orthography on L2 word form learning: (i) whether the writing system is shared by the native language and the L2, and (ii) if the writing system is shared, whether the relevant grapheme-phoneme correspondences are also shared. The acquisition of Mandarin via the *Pinyin* and *Zhuyin* writing systems provides an ecologically valid opportunity to explore these factors. We first asked whether there is a difference in native English speakers' ability to learn *Pinyin* and *Zhuyin* grapheme-phoneme correspondences. In Experiment 1, native English speakers assigned to either *Pinyin* or *Zhuyin* groups were exposed to Mandarin words belonging to one of two conditions: in the “congruent” condition, the *Pinyin* forms are possible English spellings for the auditory words (e.g., < nai> for [nai]); in the “incongruent” condition, the *Pinyin* forms involve a familiar grapheme representing a novel phoneme (e.g., < xiu> for [ɕiou]). At test, participants were asked to indicate whether auditory and written forms matched; in the crucial trials, the written forms from training (e.g., < xiu>) were paired with possible English pronunciations of the *Pinyin* written forms (e.g., [ziou]). Experiment 2 was identical to Experiment 1 except that participants additionally saw pictures depicting word meanings during the exposure phase, and at test were asked to match auditory forms with the pictures. In both experiments the *Zhuyin* group outperformed the *Pinyin* group due to the Pinyin group's difficulty with “incongruent” items. A third experiment confirmed that the groups did not differ in their ability to perceptually distinguish the relevant Mandarin consonants (e.g., [ɕ]) from the foils (e.g., [z]), suggesting that the findings of Experiments 1 and 2 can be attributed to the effects of orthographic input. We thus conclude that despite the familiarity of *Pinyin* graphemes to native English speakers, the need to suppress native language grapheme-phoneme correspondences in favor of new ones can lead to less target-like knowledge of newly learned words' forms than does learning *Zhuyin*'s entirely novel graphemes.

## Introduction

Adult second language (L2) learners can exploit the availability of orthographic input in learning the phonological forms of L2 words (e.g., Escudero et al., 2008). However, we have also seen that there are limits to the utility of orthographic input in supporting learners' target-like acquisition of words' forms—the literature provides cases where written input either had no beneficial effect (Simon et al., 2010; Hayes-Harb and Hacking, 2015; Showalter and Hayes-Harb, 2015) or in fact interfered with the target-like acquisition of L2 word forms (e.g., Hayes-Harb et al., 2010; Young-Scholten and Langer, 2015). Two factors that have emerged as possibly associated with whether or not orthographic input supports or interferes with word form learning are (i) whether the writing system is shared by the native language and the L2, and (ii) if the writing system is shared, whether the relevant grapheme-phoneme correspondences are also shared. The case of native English speakers learning Mandarin via the *Zhuyin* and *Pinyin* writing systems provides an ecologically valid opportunity to explore the relative impact of these two factors on L2 word form learning. *Pinyin* uses the Roman alphabet, shared with English, while *Zhuyin* uses an entirely different set of graphemes. Each writing system poses its own set of challenges to native English learners: *Zhuyin* requires learners to acquire an entirely novel grapheme set; *Pinyin*, on the other hand, involves only familiar graphemes, but learners must suppress a number of English grapheme-phoneme correspondences in favor of new ones (e.g., in Mandarin, *Pinyin* < x>^1^ maps to /ɕ/). In the present study we explored the consequences of these characteristics of *Pinyin* and *Zhuyin* for native English speakers' ability to learn the phonological forms of a set of Mandarin words, with the goal of elucidating the relative difficulty associated with each writing system.

## Orthographic input and L2 phonological acquisition

The role of orthographic input in L2 phonological and lexical acquisition has received increased attention in recent years (Bassetti, 2008; Bassetti et al., 2015). While a number of studies have demonstrated a facilitative effect of orthographic input for L2 learners (e.g., Escudero et al., 2008; Showalter and Hayes-Harb, 2013), others have found limited or no effect of orthographic input (e.g., Simon et al., 2010; Pytlyk, 2011; Escudero, 2015; Hayes-Harb and Hacking, 2015; Showalter and Hayes-Harb, 2015). Indeed, there are also circumstances where orthographic input can interfere with L2 phonological and lexical acquisition (Bassetti, 2006; Escudero and Wanrooij, 2010; Hayes-Harb et al., 2010; Mathieu, 2016). We begin by reviewing studies on the influence of orthographic input in L2 word form learning, followed by a discussion of the small number of studies that have considered the influence of *Zhuyin* and *Pinyin* input on L2 Mandarin phonological and lexical acquisition.

### Orthographic input and L2 word form learning

In cases where orthographic input facilitates L2 word form learning, learners may benefit from familiarity of the graphemes in addition to familiarity of the grapheme-phoneme correspondences. For example, native Dutch speakers who saw written forms during an English word learning task (e.g., < tandek> and < tenzer>) were more likely to have established lexical representations that distinguish between English /æ/ and /ɛ/ (corresponding to the letters < a> and < e>) than those who did not have access to written forms (Escudero et al., 2008). In this case, the L2 English graphemes were familiar to the native Dutch learners, and additionally, while the particular grapheme-vowel mappings differ between Dutch and English, the graphemes < a> and < e> capture a phonological contrast in both languages, presumably allowing participants to infer the English phonological contrast from the differential spellings.

More recent studies, however, have provided evidence of the limitations of written input in facilitating second language word learning. For example, a number of studies have found no effect of orthographic input in some cases where the graphemes and/or grapheme-phoneme correspondences are unfamiliar (e.g., Simon et al., 2010; Showalter and Hayes-Harb, 2013). Others have even found detrimental effects when the grapheme-phoneme correspondences of the L1 and L2 differ (Young-Scholten, 2002; Hayes-Harb et al., 2010; Hayes-Harb et al., submitted), or when the orthography is entirely unfamiliar (e.g., Mathieu, 2016). For example, Hayes-Harb et al. (2010) demonstrated that using a familiar orthography with unfamiliar grapheme-phoneme correspondences can lead learners to misremember the phonological forms of newly learned words. In this study, native English speakers were taught a set of auditory English non-words along with pictured meanings, and were later tested on their ability to match auditory forms to the pictures. In the “congruent” condition, participants always saw written forms (presented immediately below the picture) that were spelled according to English grapheme-phoneme correspondences (e.g., the auditory form [faza] was accompanied by the written form < faza>). In the “incongruent” condition, participants saw some written forms did not conform to English grapheme-phoneme correspondences (e.g., the auditory form [faza] was accompanied by < fasha>). In the control condition, participants saw < xxx> instead of written forms. At test, participants in the incongruent condition were more likely than participants in the other two conditions to misremember the phonological forms of the words in ways that reflected the (incongruent) spellings (e.g., accept [fa∫a] as a possible pronunciation of the word [faza]). In this way, the incongruent spellings of the newly learned words appear to have interfered with participants' ability to correctly remember the words' phonological forms at test. Hayes-Harb et al. (submitted), following up on earlier studies such as those of Young-Scholten (2002) and Young-Scholten and Langer (2015), demonstrated that access to spelled forms in the L2 input can interfere with native English speakers' acquisition of German final obstruent devoicing. Hayes-Harb et al. (submitted) taught native English speakers German nonwords in two conditions: in one condition, participants saw spelled forms (e.g., hear [krɑt]; see < krad>); in the other condition, participants did not see spelled forms. At test, participants who had seen < krad> during the word learning phase were more likely than those in the no spelled forms condition to pronounce it as [krɑd]. They conclude that in cases where auditory forms and written forms conflict, inferences about the pronunciation of words from written input may override the auditory input. Escudero et al. (2014) provide additional evidence that “congruency” between the grapheme-phoneme correspondences of the L1 and L2 influence the effect of written input on L2 word form learning. They taught native Spanish speakers auditory Dutch nonwords and pictured meanings in two conditions (one with and one without written forms), and later tested them on their ability to distinguish between minimal pairs of test words. In this study, “congruency” was defined somewhat differently than in other studies mentioned here in that it related to whether or not a graphemic contrast signals a phonemic contrast in both the L1 and L2, not to the grapheme-phoneme correspondences themselves. Some pairs of test words were “congruent” in the sense that the corresponding orthographic forms signal a phonemic contrast in both Spanish and English (e.g., Dutch < i> − < uu> = /i/ − /y/ and Spanish < i> − < u> = /i/ − /u/), while others were “incongruent” in that the orthographic forms signal a phonemic contrast in Dutch but not in Spanish (e.g., Dutch: < u> − < uu> = /y/ − /y/; Spanish: < u> = /u/). The native Spanish participants performed more accurately at test on congruent than incongruent items. Escudero et al. (2014) thus found further evidence that L2 learners experience a benefit associated with congruency between the L1 and L2 writing systems when learning new words.

Why should auditory and orthographic input interact in these ways in second language word learning? The influence of orthography on spoken word recognition is well documented. For example, Ziegler and Ferrand (1998) demonstrated that native French speakers respond faster in an *auditory* lexical decision task to words whose rimes have a single possible spelling (e.g., < age> for for the rime /ɑʒ/) than to words whose rimes can be spelled variously (e.g., < omb> or < om> for the rime /om/). In addition, the effect of orthography on phonological processing begins in childhood along with early literacy. For example, Racine et al. (2014) found that native French readers (9–10 years old) show evidence for the influence of words' spelled forms on their auditory processing of French production variants resulting from schwa deletion, while native French pre-readers (5–6 year olds) do not.

As noted by Veivo and Jarvikivi (2013), a “consequence of many L2 learners being literate is that the teaching and the learning of L2 are often based on written language to a significant degree” (p. 866). Thus L2 learners' (alphabetic) literacy, presumably including their knowledge of specific grapheme-phoneme correspondences and/or the expectation that written input will provide phonologically relevant information about the forms of L2 words, may exert an influence beginning with their earliest exposure to L2 words. In light of the vast literature documenting learners' propensity for transferring aspects of their L1 into L2 acquisition (see, e.g., Eckman and Iverson, 2013), it may be unsurprising that learners appear to transfer their L1 grapheme-phoneme correspondences to L2 learning.

Relative to the number of studies that have considered the impact of orthographic congruency on L2 word form learning, very few have investigated the effect of unfamiliar orthographies. Hayes-Harb and Hacking (2015) investigated the influence of diacritic stress marks on Russian written words on native English speakers' ability to learn Russian lexical stress. They secondarily asked whether the effect of stress marks differed depending on whether the words were written in Cyrillic or Roman letters. They found no beneficial effect of the diacritic stress marks, and no difference in performance associated with the Cyrillic vs. Roman letter condition, suggesting at a minimum that the familiarity of the graphemes did not influence word form learning. Showalter and Hayes-Harb (2015) similarly did not find a difference in word learning performance between groups of naïve native English speakers exposed to Arabic vs. Roman written forms when learning Arabic words minimally distinguished by the difficult velar /k/—uvular /q/ contrast. While these two studies do not indicate a word learning disadvantage associated with novel orthographies vs. familiar ones, it is worth noting that the measure of learning in both of these studies involved perceptually discriminating difficult novel phonological contrasts. In the present study, we focus not on the role of orthographic input in learners' ability to differentiate words containing difficult novel contrasts, but rather on the issues of orthographic congruency and familiarity and their effects on L2 word form learning.

In summary, the growing literature on the influence of orthographic input in L2 word form learning has highlighted two factors that may be associated with whether written input supports or interferes with word form learning: (1) whether the writing system is shared by the native language and the L2, and (2) if the writing system is shared, whether the relevant grapheme-phoneme correspondences are shared by the two languages. The acquisition of L2 Mandarin provides an opportunity to explore these factors, given that the *Pinyin* writing system involves familiar graphemes with a number of novel grapheme-phoneme correspondences, and *Zhuyin* involves an entirely new set of graphemes. The following section reviews the small number of studies that have considered the influence of these two writing systems on L2 Mandarin acquisition.

### Orthographic input and the acquisition of L2 mandarin

Chinese characters are known for their opacity in terms of grapheme-phoneme correspondences. Indeed, the phonetic component of a Chinese character provides reliable cues to the pronunciation of the character < 30% of the time (Cheng, 2012). To facilitate the learning of Chinese characters, a phonetic script that transparently presents the phonological forms of Chinese words is usually introduced to beginning learners (including both L1 and L2 learners). *Pinyin* and *Zhuyin* are the scripts that are most commonly used for this purpose. *Pinyin* (formally known as *Hanyu Pinyin*) is a Romanization system used in China and Singapore, and has been adopted by the International Organization for Standardization for the Romanization of Chinese ISO (2015). *Zhuyin* (also called *Zhuyin fuhao* or *Bopomofo*) consists of components of ancient Chinese characters, and is widely used in Taiwan. Crucially for the present purposes, while both are transparent phonographic writing systems, *Pinyin* and *Zhuyin* differ from one another in the graphemes they employ. There are also organizational differences between Pinyin, which is an alphabet, and Zhuyin, which is a semi-syllabary, or a combination of an alphabet and a syllabary; (Taylor and Taylor, 2014). Here we explore the differential effects of the two writing systems on the acquisition of Mandarin word forms by native English speakers. In particular, we ask whether the orthographic differences between *Pinyin* and *Zhuyin* influence Mandarin word learning. This question is particularly intriguing in the context of adult L2 Mandarin acquisition, because these learners are equipped with the knowledge of their L1 writing system, which may interact with the characteristics of *Pinyin* and *Zhuyin*. For example, native English-speaking learners, whose L1 employs the Roman alphabet, may find *Pinyin* less difficult than *Zhuyin* initially given the familiarity of the *Pinyin* symbols, which form a subset of the English alphabet. However, for a subset of *Pinyin* graphemes, the grapheme-phoneme correspondences differ from those of English. For example, in Chinese the *Pinyin* grapheme < x> maps to the voiceless alveopalatal fricative /ɕ/, a phoneme that does not exist in English; the same grapheme maps to /ks/ as in “tax” or /z/ as in “xylophone” in English. Thus native English speakers learning Mandarin who are exposed to *Pinyin* may benefit from the familiarity of the graphemes but experience difficulty learning novel grapheme-phoneme correspondences. In other words, native English speakers may show evidence of the negative transfer of English grapheme-phoneme correspondences when learning Mandarin with *Pinyin*.

On the other hand, no such opportunity for negative transfer is associated with *Zhuyin*, whose graphemes do not overlap with English graphemes. For instance, the voiceless alveolar affricate /ts/, which is written < z> in *Pinyin*, is written <ㄗ> in *Zhuyin*. Table 1 provides example Zhuyin and Pinyin graphemes, along with their corresponding phonemes. *Zhuyin*, however, presents its own challenge for native English speakers—that of learning a new set of graphemes. At present we are interested in the relative difficulty associated with learning new graphemes vs. learning new grapheme-phoneme correspondences on native English speakers' ability to learn the phonological forms of new Mandarin words.

**Table 1:** **Example *Pinyin* and *Zhuyin* graphemes and their corresponding Mandarin phonemes**.

***Pinyin***	***Zhuyin***	**Corresponding mandarin phoneme**
n	ㄋ	Alveolar nasal /n/
s	ㄙ	Voiceless alveolar fricative /s/
l	ㄌ	Alveolar lateral /l/
m	ㄇ	Bilabial nasal /m/
z	ㄗ	Voiceless dental affricate /ts/
c	ㄘ	Voiceless aspirated dental affricate /ts^h^/
q	ㄑ	Voiceless aspirated alveopalatal affricate /tɕ^h^/
x	ㄒ	Voiceless alveopalatal fricative /ɕ/

A small number of studies have specifically investigated the influence of orthographic input on the acquisition of Mandarin by native English speakers (Bassetti, 2006; Pytlyk, 2011; Showalter and Hayes-Harb, 2013). Bassetti (2006) and Pytlyk (2011) specifically explored the acquisition of Mandarin by native English speakers via the written medium of *Pinyin*, focusing on the potential for interference due to the negative transfer of native English grapheme-phoneme correspondences. Bassetti (2006) investigated whether *Pinyin* spelling conventions for rimes influences native English speakers' Mandarin phonological representations, focusing on the confusion they may cause for native English speakers with respect to the number of segments contained in the rimes. In *Pinyin*, rimes may be spelled differently depending on their context, in particular with respect to the inclusion of a letter representing what is called the “main vowel.” For example, following a consonantal onset, the rime /uei/ is spelled < ui> (without a letter corresponding to the main vowel /e/), as in < kui>. The same rime is spelled < wei> (with the letter < e> representing the main vowel) in onsetless syllables. Bassetti (2006) asked native English speakers who were beginning learners of Mandarin to perform two phonological tasks. In the phoneme counting task, participants were asked to read (logographic) Chinese characters and to count the number of “sounds” in each. In the phoneme segmentation task, participants were asked to pronounce the characters' sounds one-by-one. Bassetti found that for syllables where the *Pinyin* spellings do not represent the main vowel, participants counted one fewer vowel in the rime than when the *Pinyin* spellings represent the main vowel. The segmentation task confirmed that the vowel omitted by learners was indeed the main vowel, or the one that is not represented in *Pinyin* spellings. Bassetti concluded that the native English speakers' phonological representations for Chinese syllables was affected by the *Pinyin* spelling conventions with respect to main vowels.

Pytlyk (2011) investigated whether exposure to *Pinyin*, in particular in cases where English and Mandarin have different grapheme-phoneme correspondences, negatively influences native English speakers' ability to perceive Mandarin consonants. Pytlyk predicted that while native English speakers may benefit from the positive transfer of knowledge of the Roman alphabet in learning Mandarin via *Pinyin* (a “shared” orthography), they may experience difficulty where the grapheme-phoneme correspondences of *Pinyin* and the English alphabet differ. Specifically, the prediction was that “learners who learn Mandarin via Pinyin…will tend to equate a similar Mandarin (L2) phoneme with its English counterpart because the shared orthographic symbols would make perceiving the differences between the similar sounds even more difficult” (p. 545). In contrast, it was predicted that learner groups who were exposed to *Zhuyin* or to no written forms at all would outperform the *Pinyin* learners in Mandarin consonant perception because neither of these groups would experience the orthographic interference associated with *Pinyin*. Native English speakers with no previous Chinese language experience participated in a language training phase followed by a perception test. During the language training phase, they were taught the Mandarin phoneme inventory via *Pinyin, Zhuyin*, or no written input. At test, participants performed an odditiy discrimination task, in which they heard three stimuli and were asked to determine which one differed from the other two. There were no significant differences in test performance among the participants trained via *Pinyin, Zhuyin*, or no written input. While Pytlyk (2011) did not find the predicted differences in perception performance, this study nonetheless highlights the utility of Mandarin and its *Pinyin* and *Zhuyin* writing systems for addressing questions concerning the role of orthographic transfer in second language phonological learning.

### Research questions

The Bassetti (2006) and Pytlyk (2011) studies investigated the influence of orthographic input on phonological representations of Mandarin syllables and on the ability of learners to perceive Mandarin phonological contrasts, respectively. In focus in the present work is the influence of orthographic input in early lexical-phonological development—specifically, the influence of *Pinyin* and *Zhuyin* on native English speakers' ability to accurately remember the phonological forms of newly learned Mandarin words. The broadest question guiding our research is thus: Is there a difference in the difficulty associated with learning the grapheme-phoneme correspondences for novel graphemes (as in *Zhuyin*) and learning new grapheme-phoneme correspondences for familiar graphemes (as in *Pinyin*)? The first research question that this study is designed to answer is whether there is a difference in native English speakers' ability to learn *Pinyin* vs. *Zhuyin* grapheme-phoneme correspondences, specifically whether native English speakers exposed to *Pinyin* experience particular difficulty with “incongruent” grapheme-phoneme correspondences. This question is addressed in Experiment 1. Our second research question is whether there is a difference in native English speakers' ability to learn the phonological forms of new words when exposed to *Pinyin* vs. *Zhuyin*, specifically whether native English speakers exposed to *Pinyin* experience particular difficulty learning the phonological forms of words with “incongruent” spellings (Experiment 2). Our final research question, addressed in Experiment 3, is whether participants exposed to *Pinyin* vs. *Zhuyin* differ in their ability to perceive Mandarin consonant contrasts.

## Experiments

This study was carried out with approval from the University of Utah Institutional Review Board and with written informed consent from all participants.

### Participants

Thirty monolingual native English speakers were recruited from the University of Utah community and received course credit for participating in the study. All participated in all three experiments in the same order. A background questionnaire confirmed that none of the participants had previously studied Chinese, and none reported speech, language, hearing, or neurological disorders. The participants were randomly assigned to the *Pinyin* group or the *Zhuyin* group (*n* = 15 each). Each group consisted of 5 males and 10 females. The mean age in the *Pinyin* group was 23.7 years old (SD = 4.7), and the mean age in the *Zhuyin* group was 25.7 years old (SD = 8.7). Participants assigned to the *Pinyin* group reported experience with Spanish (8 participants), Japanese (2), French (2), and one each with Arabic, Latin, Korean, German, Modern Greek, Samoan, Turkish, and Swahili; two participants reported no second language experience. Participants in the *Zhuyin* group reported experience with Spanish (12), French (3), and one each had experience with Russian, Armenian, ASL, German, or Italian; two reported no second language experience.

### Materials

For the purposes of the study, we developed a set of 16 Mandarin syllables (“words”), along with their written forms in *Pinyin* and *Zhuyin* and randomly-assigned line-drawing visual referents (i.e., the words' “meanings”). The words belonged to two conditions: congruent and incongruent. In the congruent condition, the *Pinyin* forms are possible English spellings for the auditory words (e.g., < nai> for [nai]); in the incongruent condition, the *Pinyin* forms involve a familiar (English) grapheme representing a novel (Mandarin) consonant (e.g., the < x> in < xiu> for [ɕiou]). It is important to note that words are categorized as congruent and incongruent on the basis of their *Pinyin* spellings only—the novel Zhuyin graphemes are neither congruent nor incongruent from the point of view of participants. To determine the use of *Pinyin* graphemes in the congruent vs. incongruent word conditions, we first conducted a norming study. In this study, 10 native English speakers (who did not participate in the three experiments) were asked to use English graphemes to transcribe the initial consonants in 105 aurally-presented Mandarin CV syllables. The syllables were produced by a male Mandarin-English bilingual speaker reading from *Pinyin* transcriptions. Following a brief practice session using English nonwords to familiarize them with the task, the native English speakers were asked to respond to the entire block of 105 syllables, presented twice and in a different random order each time.

We calculated the percentage of participants' English letter responses that matched the *Pinyin* letters used to transcribe the initial consonants in Mandarin. For example, the auditory syllable /lin/, which is spelled with an initial < l> in *Pinyin*, was always transcribed by the native English participants with an initial < l>, and thus received a “match” score of 100%. On the other hand, the initial consonant in [tɕ^h^ie], transcribed as < q> in *Pinyin*, was transcribed by the native English participants as < ch, C, sh, t, ts>, but never as < q>, and thus received a match score of 0%. The four graphemes that received the highest match scores were selected for use in the congruent condition: < l> (100%), < m> (100%), < s> (98%), and < n> (96%). The four receiving the lowest match scores were selected for use in the incongruent condition: < c> (0%), < q> (0%), < x> (0%), and < z> (13%). [Note: Although *Pinyin* < zh> also had a low match score (5%), its corresponding Mandarin consonant phoneme had a similar response profiled to < q>, indicating that the Mandarin phonemes represented by < zh> and < q> are potentially confusable by native English speakers. For this reason, we excluded < zh> from the study materials].

We next created 16 Mandarin syllables using the Mandarin phonemes represented by the congruent and incongruent graphemes that were selected via the norming study. To control for lexical tone, all word stimuli were produced in Tone 4 (high-falling); in this tone, some of the words were actual words in Mandarin and others were nonwords; all are referred to here as “words” since our participants were unfamiliar with Mandarin. Due to restrictions on vowel distributions in Mandarin, words with initial < z, c, n, s> (/ts, ts^h^, n, s/, respectively) contained the rimes < ai> or < ao> (/ai/ or /au/), and those with initial < q, x, l, m> (/tɕ^h^, ɕ, l, m/) contained the rimes < ie> or < iu> (/iε/ or /iou/). Each of the eight initial consonants was combined with its two corresponding rimes to create the 16 Mandarin words; a full list of the words is provided in Table A1 in Appendix. These words served as the stimuli presented in the exposure, criterion, and test phases of the three experiments described below.

In addition, we created a set of 16 foil words for use in the test phases. For the incongruent condition, we chose the phonemes that the incongruent *Pinyin* graphemes usually represent in English to serve as foils. The foil phoneme for < z> and < x> is thus /z/, and the foil phoneme for < c> and < q> is thus /k/ (Note: as there is no /z/ phoneme in Mandarin, some of the words used in the study are in fact impossible in Mandarin; as a whole, the stimulus set is thus quasi-Mandarin). The foil phonemes for the congruent graphemes were selected randomly: /d/ for < n> and < l>, and /ɵ/ for < s> and < m>. Table 2 summarizes the construction of the Mandarin words and their foils. Words are categorized as congruent and incongruent on the basis of their *Pinyin* spellings only, given that the native English speakers who participated in the present experiments do not have existing grapheme-phoneme correspondences for the (unfamiliar) *Zhuyin* graphemes.

**Table 2:** **The *Pinyin* and *Zhuyin* graphemes used in the study, along with the foil phonemes assigned to each grapheme and the vowels added to create the Mandarin word stimuli**.

	**Grapheme**		**Corresponding phoneme(s)**		**Foil phoneme**	**Vowels added to create word stimuli**		
	***Pinyin***	***Zhuyin***	**Mandarin**	**English**		**Vowel**	***Pinyin***	***Zhuyin***
Congruent items	n	ㄋ	/n/	/n/	/d/	/ai/	ai	ㄞ
	s	ㄙ	/s/	/s/	/ɵ/	/au/	ao	ㄠ
	l	ㄌ	/l/	/l/	/d/	/iε/	ie	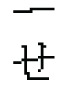
	m	ㄇ	/m/	/m/	/ɵ/	/iou/	iu	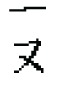
Incongruent items	z	ㄗ	/ts/	/z/	/z/	/ai/	ai	ㄞ
	c	ㄘ	/ts^h^/	/k/, /s/	/k/	/au/	ao	ㄠ
	q	ㄑ	/tɕ^*h*^/	/k/	/k/	/iε/	ie	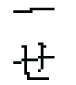
	x	ㄒ	/ɕ/	/z/, /ks/	/z/	/iou/	iu	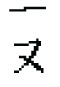

Each of the 16 words was randomly assigned a “meaning” from among a set of nonobject line drawings; the word-meaning pairings were the same for all participants. The words were produced by a male Mandarin-English bilingual speaker reading from *Pinyin* transcriptions.

### Experiment 1 (grapheme-phoneme correspondence learning) procedures

In Experiment 1, we exposed participants to the set of auditory Mandarin words and their written forms, and later tested them on their ability to accurately determine whether the auditory and written forms were correctly matched. The experiment involved three phases: exposure, criterion, and test. All three experiments were conducted in a sound-attenuated booth; the entire session lasted ~ 1 h, with brief participant-controlled breaks between experiments.

#### Exposure phase

Participants were asked to learn the 16 words. in each exposure trial, a written form was presented on the computer screen while the auditory word was played over headphones at a comfortable listening level. The written form remained on the screen for 2 s, followed by the next trial. The 16 words constituted one block, and there were four blocks in the exposure phase. see Table 3 for example exposure phase trials.

Table 3**Experiment 1 example stimuli, by phase**.

**Table:** 

**EXPOSURE PHASE**						
**Exposure condition**	**Example congruent trials**			**Example incongruent trials**		
	**See**	**Hear**	**See**	**Hear**
*Pinyin*	nai	[nai]	xiu	[ɕiou]
*Zhuyin*	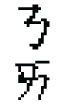		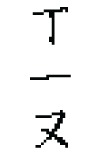

**Table:** 

**CRITERION TEST PHASE**						
**Exposure condition**	**Example congruent trials**			**Example incongruent trials**		
	**See**	**Matched hear**	**Mismatched hear**	**See**	**Matched hear**	**Mismatched hear**
*Pinyin*	nai	[nai]	[ts^h^ai]	xiu	[ɕiou]	[miou]
*Zhuyin*	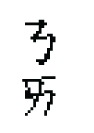			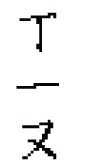

**Table:** 

**FINAL TEST PHASE**						
**Exposure condition**	**Example congruent trials**			**Example incongruent trials**		
	**See**	**Matched hear**	**Mismatched hear**	**See**	**Matched hear**	**Mismatched hear**
*Pinyin*	nai	[nai]	[dai]	xiu	[ɕiou]	[ziou]
*Zhuyin*	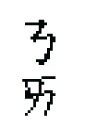			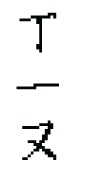		

#### Criterion phase

The criterion phase consisted of 16 matched and 16 mismatched trials. Participants were asked to indicate whether a written word matched the auditory word by pressing “yes” or “no” buttons on the keyboard. Table 3 illustrates example criterion phase trials. Congruent-matched and congruent-mismatched trials were expected to be easy for participants (e.g., see pinyin/zhuyin written form for [nai] and hear [nai] (matched) or [ts^h^ai] (mismatched)). In incongruent-matched trials, participants saw a written form and heard its corresponding auditory word (e.g., see the pinyin/zhuyin written form for the word [ɕiou] and hear [ɕiou]). In the incongruent-mismatched trials, as in the congruent-mismatched trials, participants saw a written form and heard a word beginning with an entirely different consonant that was also not the foil (e.g., see the pinyin/zhuyin written form for the word [ɕiou] but hear [miou]). In this way, no criterion phase trials were designed to be difficult for participants in either exposure condition; rather, the criterion phase was used to ensure that all participants achieved a similar level of ability to distinguish learned forms from quite different foils before continuing to the test phase. Participants repeated the exposure and criterion phases until they reached 90% accuracy on the criterion test.

#### Test phase

The test phase was identical to the criterion phase except that the test phase was designed to determine whether participants experienced confusion due to differences between Pinyin and English grapheme-phoneme correspondences. Congruent-matched and congruent-mismatched trials were again expected to be easy for participants (e.g., see Pinyin/Zhuyin written form for [nai] and hear [nai] (matched) or [dai] (mismatched)), as were the incongruent-matched trials (e.g., see the Pinyin/Zhuyin written form for the word [ɕiou] and hear [ɕiou]). However, the incongruent-mismatched trials were designed to be difficult for participants in the Pinyin condition if they experienced interference from English grapheme-phoneme correspondences. In these trials, participants saw a written form and heard a word beginning with a consonant reflecting English grapheme-phoneme correspondences (e.g., see the Pinyin/Zhuyin written form for the word [ɕiou], which is spelled < xiu> in Pinyin, but hear [ziou], a possible English pronunciation of the Pinyin written form). See Table 3 for an illustration of test phase trials.

#### Results

The first analysis concerns the number of exposure-criterion phase cycles that participants required to reach the criterion necessary to continue to the final test. Participants in the *Pinyin* group (mean = 1.6 cycles; SD = 0.632) required significantly fewer cycles than did participants in the *Zhuyin* group [mean = 3.47; SD = 1.807; *F*_(1, 28)_ = 14.255, *p* = 0.001, *partial* η^2^ = 0.337].

**Figure 1:**
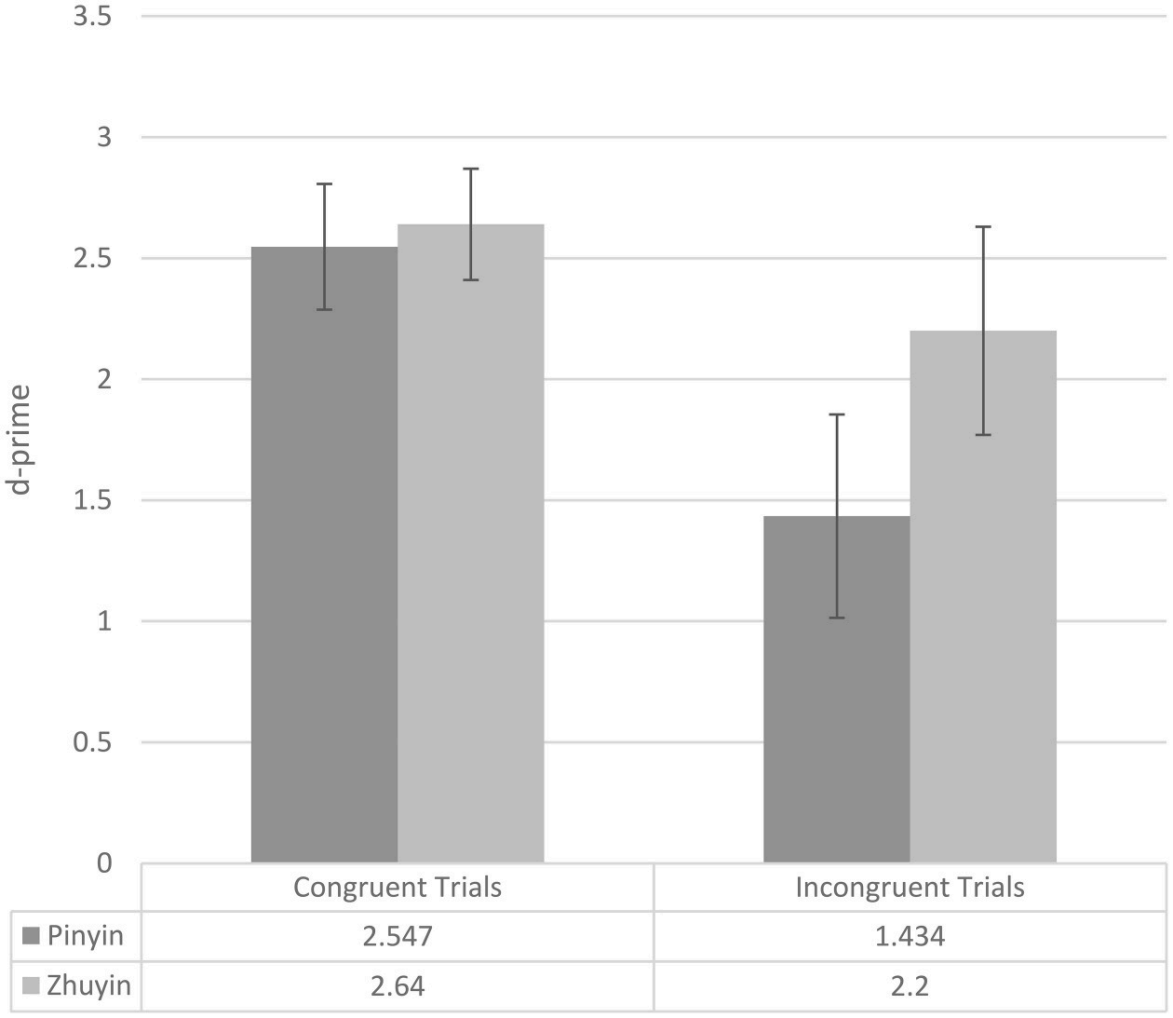
**Experiment 1 mean test d-primes (whiskers represent 95% confidence intervals), by exposure condition and item condition**.

We converted the final test phase accuracy data (see Table 4) to d-primes using Signal Detection Theory (see Figure 1; for more information about d-prime, please see MacMillan and Creelman, 2004). The d-primes were submitted to ANOVA with exposure condition (two levels: *Pinyin, Zhuyin*) as a between-subjects variable and item condition (congruent, incongruent) as a within-subjects variable. There was a main effect of exposure group, with participants in the *Zhuyin* group performing more accurately than participants in the *Pinyin* group overall [*F*_(1, 28)_ = 4.275, *p* = 0.048, *partial* η^2^ = 0.132], a main effect of item type, with higher d-primes on congruent than incongruent items [*F*_(1, 28)_ = 32.027, *p* < 0.0005, *partial* η^2^ = 0.534], and an interaction of the two [*F*_(1, 28)_ = 5.991, *p* = 0.021, *partial* η^2^ = 0.176]. Following up on the interaction, we looked at the effect of exposure condition in the two item conditions separately. On congruent items, there was no effect of exposure condition [*F*_(1, 28)_ = 0.284, *p* = 0.598, *partial* η^2^ = 0.010]. However, on incongruent items, the effect of exposure condition was significant [*F*_(1, 28)_ = 6.277, *p* = 0.018, *partial* η^2^ = 0.183], with participants in the *Zhuyin* condition outperforming those in the *Pinyin* condition^2^.

**Table 4:** **Experiment 1 test accuracy (proportion correct responses; 95% confidence intervals in parentheses), by exposure condition and item condition**.

**Exposure condition**	**Congruent trials**		**Incongruent trials**	
	**Matched**	**Mismatched**	**Matched**	**Mismatched**
*Pinyin*	0.975 (0.03)	0.867 (0.07)	0.933 (0.06)	0.533 (0.12)
*Zhuyin*	0.942 (0.04)	0.925 (0.05)	0.858 (0.05)	0.867 (0.10)

### Experiment 2 (word learning) procedures

Our second research question concerned whether there is a difference in native English speakers' ability to learn the phonological forms of new words when exposed to *Pinyin* vs. *Zhuyin*. Experiment 2 was identical to Experiment 1 except that participants additionally saw line drawings depicting word meanings during the exposure phase, and at test were asked to match auditory forms with the line drawings.

#### Exposure phase

Participants were asked to learn the 16 auditory words and their pictured meanings. For each word, a written word form, a picture representing the word meaning, and an auditory word were presented simultaneously and stayed on the screen for 4 s, followed by the next trial. The 16 words constituted one block, and there were four blocks in the exposure phase.

#### Criterion phase

The criterion phase trials were identical to those in Experiment 1 except that instead of matching auditory words to written forms, participants were asked to determine the accuracy of the match between auditory words and pictures. Again, congruent-matched and congruent-mismatched trials were expected to be easy for participants (e.g., see the picture associated with the auditory word [nai] and hear [nai] (matched) or [ts^h^ai] (mismatched)). In incongruent-matched trials, e.g., participants saw the picture associated with the auditory word [ɕiou] and heard [ɕiou]. In the incongruent-mismatched trials, e.g., participants saw the picture associated with the auditory word [ɕiou] but heard [miou]. Participants repeated the exposure and criterion phases until they reached 90% accuracy on the criterion test.

#### Test phase

The test phase trials were identical to those in Experiment 1, again with the exception that participants' task was to determine the accuracy of the match between auditory words and pictures. Congruent-matched and congruent-mismatched trials involved, e.g., seeing the picture associated with [nai] and hearing [nai] (matched) or [dai] (mismatched). Incongruent-matched trials involved, e.g., seeing the picture associated with [ɕiou] and hearing [ɕiou]. In incongruent-mismatched trials, participants saw a picture associated with, e.g., [ɕiou], but heard [ziou], a possible english pronunciation of the pinyin written form < xiu>. Table 5 illustrates the stimuli encountered during the exposure, criterion, and final test phases in Experiment 2.

Table 5**Experiment 2 example stimuli, by phase**.

**Table:** 

**EXPOSURE PHASE**				
**Exposure condition**	**Example congruent trials**		**Example incongruent trials**	
	**See**	**Hear**	**See**	**Hear**
	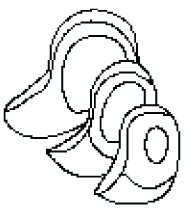		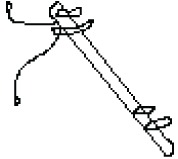	
*Pinyin*	nai	[nai]	xiu	[ɕiou]
*Zhuyin*	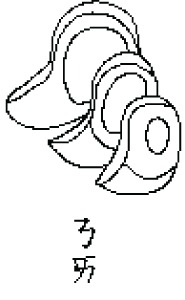		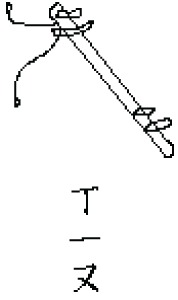

**Table:** 

**CRITERION TEST PHASE**						
**Exposure condition**	**Example congruent trials**			**Example incongruent trials**		
	**See**	**Matched hear**	**Mismatched hear**	**See**	**Matched hear**	**Mismatched hear**
*Pinyin* *Zhuyin*	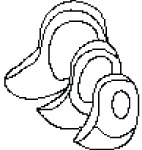	[nai]	[ts^h^ai]	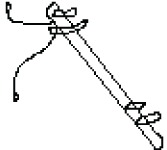	[ɕiou]	[miou]

**Table:** 

**FINAL TEST PHASE**						
**Exposure condition**	**Example congruent trials**			**Example incongruent trials**		
	**See**	**Matched hear**	**Mismatched hear**	**See**	**Matched hear**	**Mismatched hear**
*Pinyin* *Zhuyin*	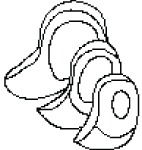	[nai]	[dai]	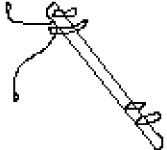	[ɕiou]	[ziou]

#### Results

**Figure 2:**
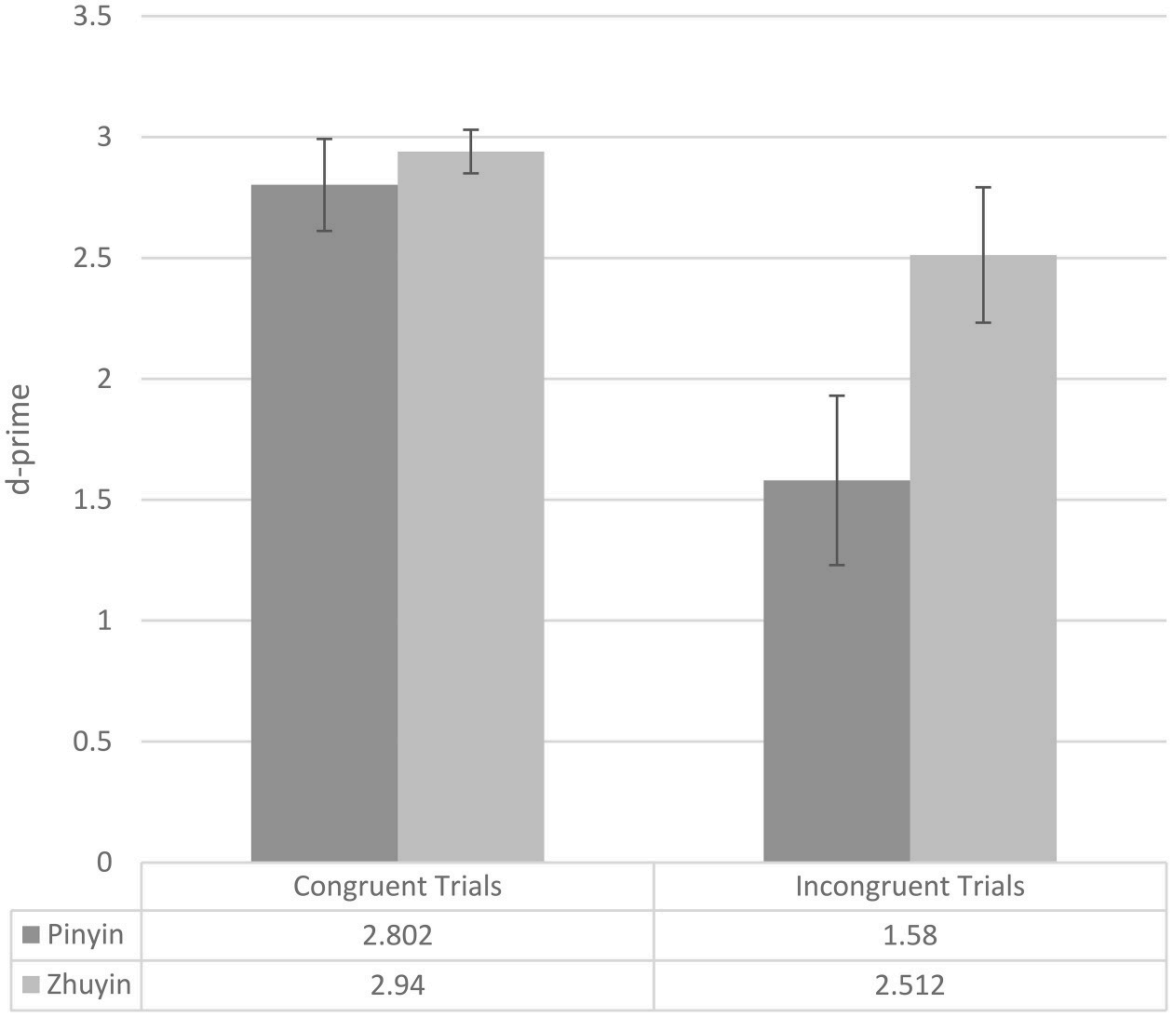
**Experiment 2 mean test d-primes (whiskers represent 95% confidence intervals), by exposure condition and item condition**.

Again, we first consider the number of exposure-criterion phase cycles participants in the two exposure conditions required. In this experiment, participants in the *Pinyin* group (mean = 2.53; SD = 0.834) required on average more cycles than did participants in the *Zhuyin* group (mean = 2.00; SD = 0.655); however, this difference was only marginally significant [*F*_(1, 28)_ = 3.797, *p* = 0.061, *partial* η^2^ = 0.119]. Table 6 presents the final test accuracy data and Figure 2 the d-primes. The d-primes were submitted to ANOVA with exposure condition (two levels: *Pinyin, Zhuyin*) as a between-subjects variable and item condition (congruent, incongruent) as a within-subjects variable. There was a main effect of exposure group, with participants in the *Zhuyin* group performing more accurately than participants in the *Pinyin* group overall [*F*_(1, 28)_ = 14.410, *p* = 0.001, *partial* η^2^ = 0.340], a main effect of item type, with higher d-primes on congruent than incongruent items [*F*_(1, 28)_ = 56.571, *p* < 0.0005, *partial* η^2^ = 0.669], and an interaction of the two [*F*_(1, 28)_ = 2.362, *p* = 0.001, *partial* η^2^ = 0.318]. Following up on the interaction, we looked at the effect of exposure condition in the two item conditions separately. On congruent items, there was no effect of exposure condition [*F*_(1, 28)_ = 1.688, *p* = 0.204, *partial* η^2^ = 0.056]. However, on incongruent items, the effect of exposure condition was significant [*F*_(1, 28)_ = 32.027, *p* < 0.0005, *partial* η^2^ = 0.534], with participants in the *Zhuyin* condition outperforming those in the *Pinyin* condition.

**Table 6:** **Experiment 2 test accuracy (proportion correct responses; 95% confidence intervals in parentheses), by exposure condition and item condition**.

**Exposure condition**	**Congruent trials**		**Incongruent trials**	
	**Matched**	**Mismatched**	**Matched**	**Mismatched**
*Pinyin*	0.975 (0.03)	0.942 (0.06)	0.850 (0.06)	0.683 (0.12)
*Zhuyin*	0.967 (0.03)	0.992 (0.02)	0.900 (0.05)	0.925 (0.07)

### Experiment 3 (consonant discrimination) procedures

The purpose of Experiment 3 was to determine whether the participants in the *Pinyin* group and those in the *Zhuyin* group differed in their ability to perceptually distinguish the consonants contained in the newly learned words from the foil consonants contained in the incongruent-mismatched trials. Because the foil consonants (e.g., [z]) were sometimes phonetically similar to the relevant Mandarin consonants (e.g., [ɕ]), performance in the test phase may have been confounded by perceptual confusability, which would undermine our ability to attribute Experiments 1 and 2 performance to the influence of the written input. Experiment 3 involved 16 matched and 16 mismatched trials. In each trial, two auditory words were presented, and participants were asked to decide whether the two words that they heard were the same. In the matched trials, each of the 16 words was presented twice. In the mismatched trials, each of the 16 words was presented with its foil (from Experiments 1 and 2; see Table A1 in Appendix for each word's foil).

#### Results

**Figure 3:**
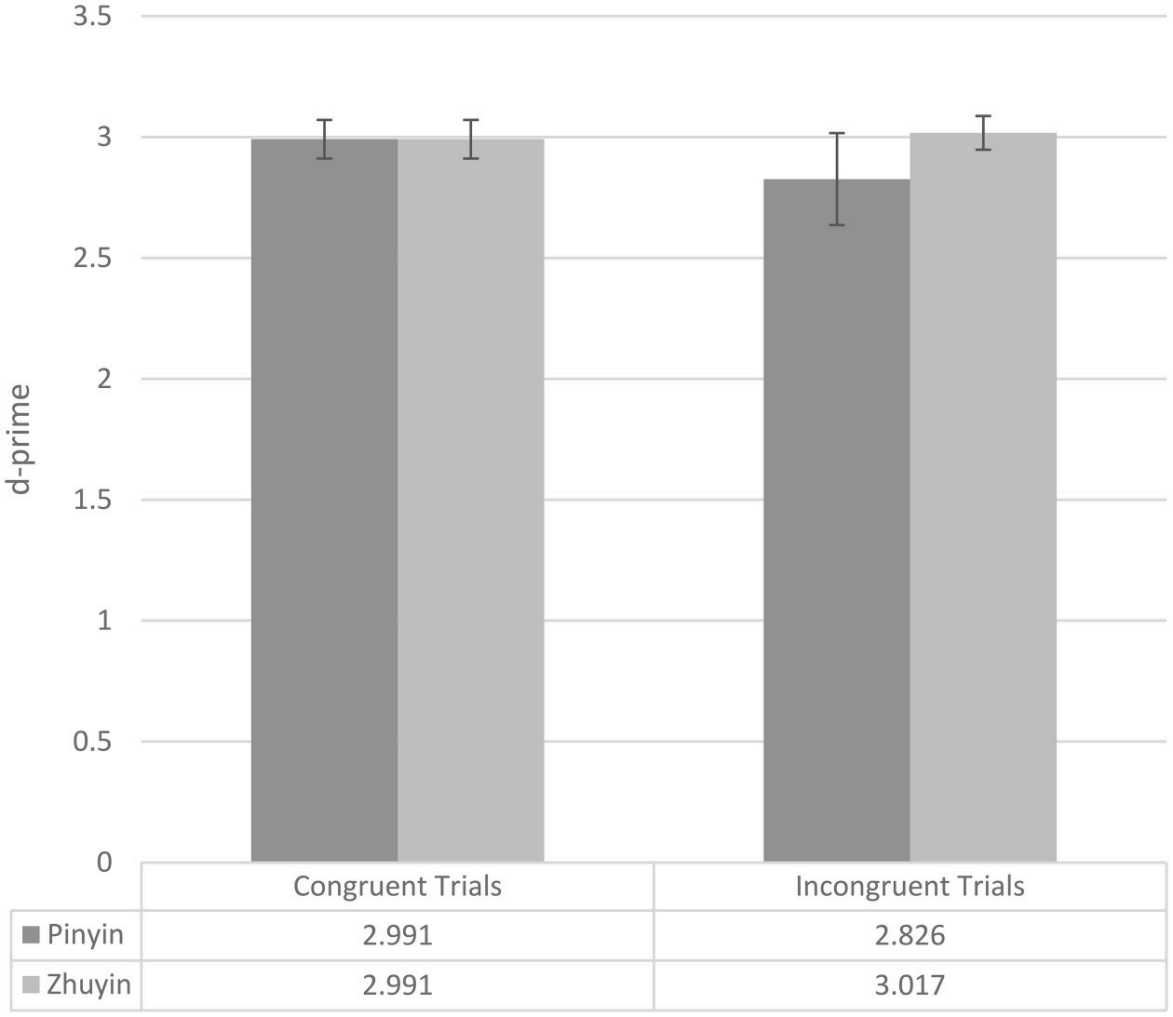
**Experiment 3 mean test d-primes (whiskers represent 95% confidence intervals), by exposure condition and item condition**.

In this final experiment, participants were tested on their ability to discriminate the Mandarin consonant contrasts. As seen in Table 7 and Figure 3, participants in both groups were near ceiling in their discrimination ability. The d-primes were submitted to ANOVA with exposure condition (two levels: *Pinyin, Zhuyin*) as a between-subjects variable and item type (congruent, incongruent) as a within-subjects variable. There was no significant main effect of either exposure condition [*F*_(1, 28)_ = 2.683, *p* = 0.113, *partial* η^2^ = 0.087] or item condition [*F*_(1, 28)_ = 1.357, *p* = 0.254, *partial* η^2^ = 0.046], and the interaction was also nonsignificant [*F*_(1, 28)_ = 2.529, *p* = 0.123, *partial* η^2^ = 0.083]. Thus any differences in performance between the two groups on Experiments 1 and 2 is not attributable to differences in the two groups' perceptual sensitivities to the Mandarin consonant contrasts.

**Table 7:** **Experiment 3 accuracy (proportion correct responses; 95% confidence intervals in parentheses), by exposure condition and item condition**.

**Exposure condition**	**Congruent trials**		**Incongruent trials**	
	**Matched**	**Mismatched**	**Matched**	**Mismatched**
*Pinyin*	0.975 (0.03)	1.000 (0.00)	0.992 (0.02)	0.933 (0.05)
*Zhuyin*	0.975 (0.03)	1.000 (0.00)	0.992 (0.02)	0.975 (0.03)

## Discussion

Recall that we first asked whether there is a difference in native English speakers' ability to learn *Pinyin* and *Zhuyin* grapheme-phoneme correspondences, specifically whether native English speakers exposed to *Pinyin* experience particular difficulty with “incongruent” grapheme-phoneme correspondences. Experiment 1 was designed to address this question. Analysis of the number of exposure-criterion phase cycles required to reach the 90% accuracy criterion indicates that participants exposed to *Zhuyin* required more than twice as many cycles as did those exposed to *Pinyin*. However, on the final test, those exposed to *Zhuyin* did not experience interference from English grapheme-phoneme correspondences on the “incongruent” items, as did participants exposed to *Pinyin*. Thus while participants initially required more exposure to *Zhuyin* than to *Pinyin* to learn the grapheme-phoneme correspondences, they ultimately were able to avoid difficulty associated with the negative transfer of native language grapheme-phoneme correspondences.

We next asked whether there is a difference in native English speakers' ability to learn the phonological forms of new words when exposed to *Pinyin* vs. *Zhuyin*, specifically whether native English speakers exposed to *Pinyin* experience particular difficulty learning the phonological forms of words with “incongruent” spellings. In Experiment 2, which immediately followed Experiment 1, we examined the word learning ability of participants exposed to *Zhuyin* vs. *Pinyin* written forms. In the exposure phase of this experiment, participants heard auditory forms and saw pictures indicating the words' meanings. The pictures were accompanied by either the *Zhuyin* written form or the *Pinyin* written form. As in Experiment 1, we were interested in whether those in the *Pinyin* group would experience interference from English grapheme-phoneme correspondences on words in the incongruent condition. Indeed, at test, participants in the *Pinyin* group incorrectly accepted auditory forms reflecting English grapheme-phoneme correspondences (the foils) as the labels for newly learned words (e.g., they indicated that [ziou] was a correct pronunciation for a picture they had learned was pronounced [ɕiou], presumably due to its *Pinyin* spelling < xiu>) significantly more often than did those in the *Zhuyin* group, while there was no difference in performance between groups on words in the congruent condition.

It is interesting to note, however, that in Experiment 2, the pattern with respect to the number of exposure-criterion phase cycles required by the two groups was opposite that observed in Experiment 1. In Experiment 2, the *Pinyin* group in fact required *more* exposure-criterion phase cycles than did the *Zhuyin* group, though this difference only approached significance at *p* = 0.061. Thus the learning speed disadvantage experienced by *Zhuyin* participants in Experiment 1 (when learning grapheme-phoneme correspondences and not word meanings) did not persevere into the word learning experiment. One might intuitively anticipate initial difficulty associated with exposure to unfamiliar graphemes—indeed, in a similarly-structured study of native English speakers learning of Arabic words, Showalter and Hayes-Harb (2015) hypothesized that the unfamiliarity of the Arabic script and its conventions may have been responsible for low test accuracy levels. However, in a follow-up experiment, when the Arabic letters were replaced with Roman transliteration, they saw no increase in word learning accuracy, indicating that difficulty associated with the novel symbols was not fully responsible for the observed test difficulty. In another similarly-structured study, Hayes-Harb and Hacking (2015) did not find substantial differences in either number of exposure-criterion phase cycles or in final test accuracy between native English speakers exposed to Russian words spelled in Cyrillic vs. Roman letters. Together, the present findings, in addition to those of these Arabic and Russian studies, do not provide evidence of an initial learning detriment associated with the presence of novel graphemes in the visual input during word learning. We in fact see evidence to the contrary in the present study: relative to (familiar but incongruent) *Pinyin*, exposure to (unfamiliar) *Zhuyin* ultimately afforded a word form learning advantage. We have thus provided additional evidence for the detrimental effects of orthographic incongruency between the L1 and L2, consistent with the findings of a number of earlier studies (e.g., Hayes-Harb et al., 2010; Escudero et al., 2014).

Our final research question was whether there is a difference in native English speakers' ability to perceive Mandarin consonant contrasts when exposed to *Pinyin* vs. *Zhuyin*. Experiment 3 was designed to determine whether any differential perceptual sensitivity to the Mandarin consonant contrast existed between the two groups of participants that might undermine the interpretation of the results of Experiments 1 and 2. However, there was no effect of exposure group on perceptual sensitivity to the distinction between the consonants contained in the newly learned words and their foils, confirming that the differential performance by the two exposure groups in Experiments 1 and 2 are attributable to *Pinyin* vs. *Zhuyin* exposure rather than to differences between the groups of participants in auditory discrimination ability. It is worth noting that our finding that differences in orthographic experience of participants in the two groups did not lead to a differential ability to perceive the consonant contrasts is consistent with the findings reported by Pytlyk (2011). We thus provide evidence that incongruencies between the L1 and L2 grapheme-phoneme correspondences can impact participants' memory for words' phonological forms in the absence of impacting their perceptual sensitivity to the relevant novel phonological contrasts. This suggests that, at least under the circumstances of the present study, the difficulty associated with suppressing native language grapheme-phoneme correspondences in favor of new ones played out at the level of the lexicon, with conflicts between orthographic and phonological information often resolved in favor of orthography, which was, crucially, interpreted via grapheme-phoneme correspondence rules transferred from the native language.

## Conclusion

The study of orthographic input in L2 phonological and word form acquisition has emerged only recently, and the present study represents an additional step in the direction of understanding the specific circumstances under which L2 learners' lexical development is helped or hindered by written input. Our aim was to investigate the influence of two factors that may moderate the influence of written input on L2 word form learning: (i) whether the writing system is shared by the native language and the L2, and (ii) if the writing system is shared, whether the relevant grapheme-phoneme correspondences are also shared. We did so via a series of experiments in which native English speakers were exposed to Mandarin words via auditory and visual (picture, written) input. Native speakers of English who had access to *Pinyin* (familiar writing system, some unfamiliar grapheme-phoneme correspondences) experienced difficulty learning the words' phonological forms due to interference from English grapheme-phoneme correspondences. Those who had access to *Zhuyin* (unfamiliar writing system) experienced no such interference, though they did initially take somewhat longer to learn the words' written forms.

In light of the fact that both *Pinyin* and *Zhuyin* are used in pedagogical settings to support Mandarin language acquisition, our findings can contribute to an understanding of the costs and benefits of each for this purpose. In particular, given that literate L2 learners are likely, especially in instructed settings, to be exposed to new words' phonological forms and their written forms more or less simultaneously, it is crucial that we understand the ways in which these two types of input impact the establishment and subsequent use of L2 lexical representations. Short laboratory-based studies like the one presented here differ importantly from real-world language acquisition; however, they do permit us to isolate and examine the factors that may contribute to L2 learning success or difficulty. One might next ask whether the patterns identified in the present study with respect to *Pinyin*'s and *Zhuyin*'s influence on L2 word form learning play out in actual native English-speaking learners of Mandarin, and whether Mandarin language experience (see Veivo and Jarvikivi, 2013) or other factors can moderate the effects of orthographic input.

## Author contributions

RH and HC collaborated on this project while HC was a post-doctoral researcher under the supervision of RH. RH and HC were involved at all stages of the project, from its conception through design, data collection, and analysis. RH was responsible for preparing the manuscript for publication, in consultation with HC.

### Conflict of interest statement

The authors declare that the research was conducted in the absence of any commercial or financial relationships that could be construed as a potential conflict of interest.

## Footnotes

^1^“ < >” denotes a written form.

^2^At the suggestion of an anonymous reviewer, we explored whether the English word status of the letter sequence < lie> (relative to the English nonword status of all other words' spelled forms) may have impacted performance on items involving < lie>. Analysis of proportion correct scores among Pinyin group participants in Experiment 1 indicates that < lie> items elicited accuracy within the range of that of the other items.

## Appendix

**Table A1:** **Complete list of Mandarin words' auditory forms, their written forms in *Pinyin* and *Zhuyin*, their auditory foils, and their pictured meanings**.

	***Pinyin***	***Zhuyin***	**Auditory form**	**Auditory foil**	**Pictured meaning**
Congruent items	nai		/naɪ/	/daɪ/	
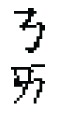
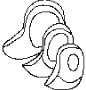
	nao		/nau/	/dau/	
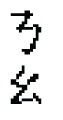
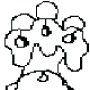
	sai		/saɪ/	/ɵaɪ/	
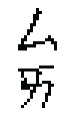
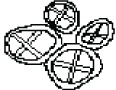
	sao		/sau/	/ɵau/	
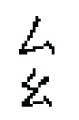
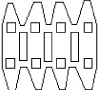
	lie		/liɛ/	/diɛ/	
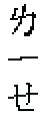
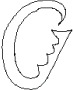
	liu		/liou/	/diou/	
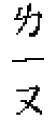
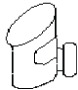
	mie		/miɛ/	/ɵiɛ/	
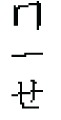
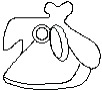
	miu		/miou/	/ɵiou/	
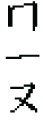
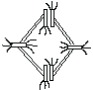
Incongruent items	zai		/tsaɪ/	/zaɪ/	
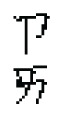
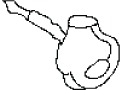
	zao		/tsau/	/zau/	
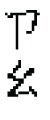
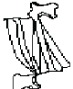
	cai		/ts^h^aɪ/	/kaɪ/	
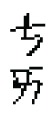
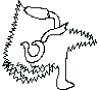
	cao		/ts^h^au/	/kau/	
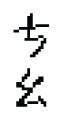
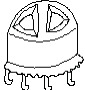
	qie		/tɕ^h^iɛ/	/kiɛ/	
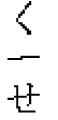
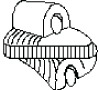
	qiu		/tɕ^h^iou/	/kiou/	
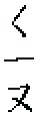
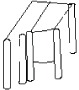
	xie		/ɕiɛ/	/ziɛ/	
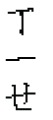
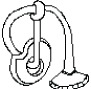
	xiu		/ɕiou/	/ziou/	
